# Fatal Powassan virus encephalitis in patients with chronic lymphocytic leukemia

**DOI:** 10.1038/s41408-022-00737-y

**Published:** 2022-10-07

**Authors:** Isla M. Johnson, Caleb Scheckel, Sameer A. Parikh, Mark Enzler, Jennifer Fugate, Timothy G. Call

**Affiliations:** 1 Department of Internal Medicine, Mayo Clinic, Rochester, MN USA; 2 Division of Hematology, Mayo Clinic, Rochester, MN USA; 3 Division of Infectious Diseases, Mayo Clinic, Rochester, MN USA; 4 Department of Neurology, Mayo Clinic, Rochester, MN USA

**Keywords:** Chronic lymphocytic leukaemia, Infectious diseases

Dear Editor,

Patients with chronic lymphocytic leukemia (CLL) are at increased risk for infection due to immunosuppression that is both intrinsic to the disease and secondary to therapy. Although bacterial infections are the most common, CLL results in T-cell dysfunction that also predisposes patients to a wide variety of viral infections [1]. Treatment with chemoimmunotherapy, Bruton tyrosine kinase inhibitors (BTKi), and B-cell lymphoma 2 inhibitors (BCL2i) can also exacerbate immune dysfunction leading to an increased risk of opportunistic infections. Common viral infections in CLL patients include herpes simplex, herpes zoster, Epstein-Barr and cytomegalovirus, and more recently, severe acute respiratory syndrome coronavirus-2 [2, 3].

Due to this immunosuppression, CLL patients may be vulnerable to severe infection from other opportunistic pathogens, such as endemic flaviviruses, that typically cause minimal symptoms in immunocompetent individuals. Powassan virus is an arthropod-borne flavivirus that is transmitted to humans primarily by the *Ixodes scapularis* tick. Although this tick is more well-known for transmitting *Borrelia burgdorferi* and *Anaplasma*, it can also transmit the Powassan virus and is endemic to the Great Lakes region and the Northeastern United States. When severe, infection with Powassan virus can cause meningoencephalitis with a case fatality rate of 10%. Approximately half of the people who survive severe disease can experience permanent neurologic sequalae [4]. We present two cases of CLL patients who contracted the Powassan virus and later died due to complications related to this disease.

Case 1: A 68-year-old male with a history of relapsed high-risk CLL (del17p), receiving therapy with the BTKi ibrutinib, was transferred to our institution after presenting with headache, recurrent fever, and rapidly progressive weakness following a recent hunting trip where he noticed a tick bite. He had recently completed a 10-day course of doxycycline for presumed Rocky Mountain Spotted Fever without clinical improvement. He experienced progressive ascending flaccid paralysis with bulbar weakness and involuntary head and neck movements, requiring intubation for airway protection and transfer to our institution. A lumbar puncture on admission demonstrated a lymphocytic pleocytosis with elevated opening pressure of 27 cm H_2_O and a negative infectious workup (Table 1). He was diagnosed initially with Guillain-Barre syndrome and received intravenous immunoglobulin (IVIG) without clinical improvement; immunoglobulin levels prior to IVIG were not available. The patient did not have known hypogammaglobulinemia requiring IVIG replacement therapy prior to admission. MRI brain was consistent with cerebellar cerebritis without hydrocephalus (Fig. 1a). Cerebrospinal fluid (CSF) IgM serology ultimately was positive for Powassan virus by enzyme-linked immunosorbent assay (ELISA), which led to a Powassan virus encephalitis diagnosis. Ibrutinib was held early in admission due to concerns that it was contributing to immune suppression and worsening of his viral infection. Immunoglobulin G (IgG) levels were initially normal at 1010 mg/dL early during the course of his hospitalization shortly after receiving IVIG but then decreased to 556 mg/dL (Table 1). Due to this borderline hypogammaglobinemia and a lack of clinical improvement, he received additional IVIG without meaningful change in his clinical status. The patient subsequently developed high fevers, pancytopenia, and organomegaly. Bone marrow biopsy revealed diffuse large B-cell lymphoma, which concerned Richter’s transformation. The possibility of Richter’s transformation further worsened this patient’s prognosis, especially as ibrutinib and high-dose steroids were started without improvement in mental status. Given continued clinical deterioration due to likely Richter’s transformation and Powassan virus encephalitis, the patient was transitioned to comfort care.

**Table 1:** Patient characteristics of two patients with CLL who contracted fatal Powassan encephalitis.

	Patient 1	Patient 2
Age	68	75
Sex	Male	Male
Duration of ibrutinib therapy	3 years, 10 months	8 years, 6 months
Number and type of prior treatments	Rituximab/pentostatin/cyclophosphamide	Fludarabine/cyclophosphamide Fludarabine /cyclophosphamide/Oblimersen Rituximab/pentostatin/cyclophosphamide
CBC with differential	WBC 11.9 × 109/L (10.05 neutrophils, 1.27 lymphocytes, 0.57 monocytes, <0.03 eosinophils, 0.05 basophils) Hb 12 gm/dL Plt 159 × 109/L	WBC 6.7 × 109/L (5.57 neutrophils, 1.3 lymphocytes, 0.52 monocytes, 0.1 eosinophils, <0.03 basophils) Hb 15.1 gm/dL Plt 164 × 109/L
Serum immunoglobulin levels	IgA 63 mg/dL IgM 35 mg/dL IgG 556 mg/dL	IgG 416 mg/dL (received IVIG every 6 weeks prior to admission)
CSF findings	Total nucleated cells: 274 (86% lymphocytes). Protein: 84 mg/dl. Glucose: 107 mg/dL. Cytology: small population of abnormal small to intermediate-size lymphocytes	Total nucleated cells: 76 (89% lymphocytes. Protein: 34 mg/dl. Glucose: 64 mg/dL. Cytology: minimal involvement with known CLL clone with reactive immunoblasts

*WBC* white blood cells, *Hb* hemoglobin, *Plt* platelets.

**Fig. 1:**
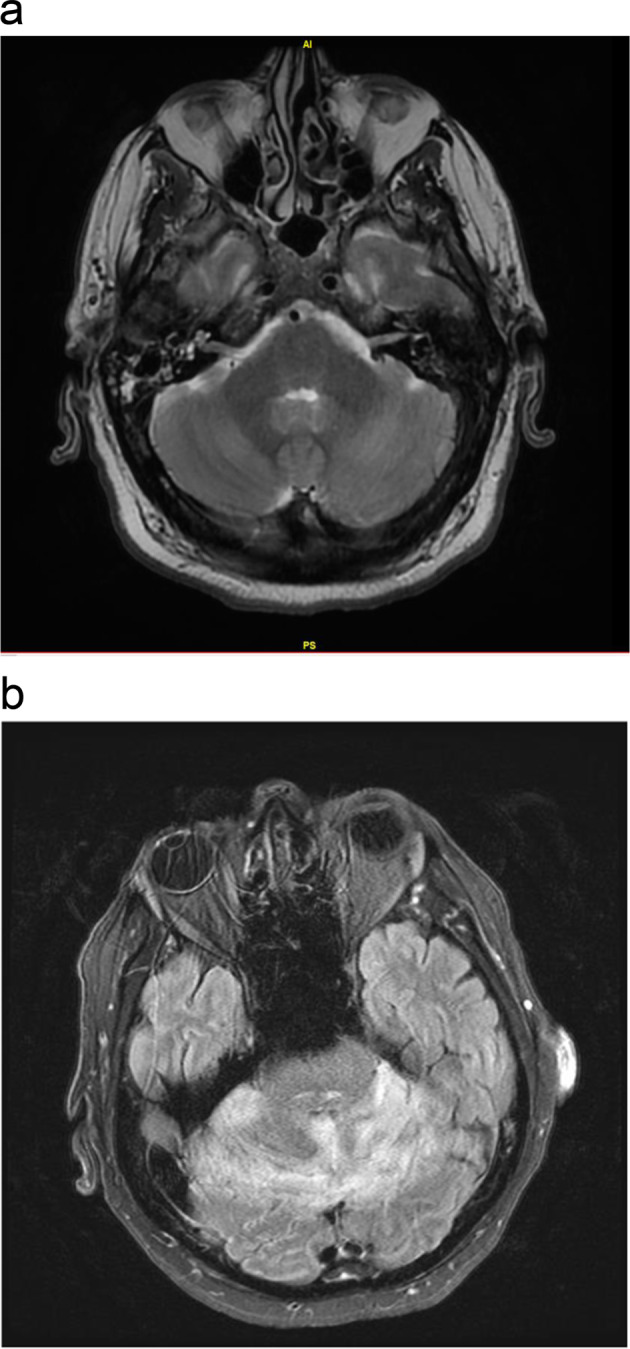
Demonstrates MRI findings of neurologic involvement in Powassan virus encephalitis. **a** Axial T2 weighted MRI shows T2 signal abnormality in the cerebellum consistent with cerebritis in patient 1. **b** Axial FLAIR image shows T2 signal abnormality consistent with cerebellar swelling in patient 2.

Case 2: A 75-year-old man with Parkinson’s disease and relapsed high-risk CLL (del17p), on ibrutinib therapy, was transferred to our institution for headache and encephalopathy. Similar to case 1, he had recently suffered a tick bite and had been treated with an outpatient course of doxycycline without clinical improvement. Upon transfer, his exam demonstrated rigidity with saccades and vertical upgaze restriction. Carbidopa/levodopa was uptitrated given concern for progressive supranuclear palsy, but he continued to decline. MRI brain, obtained following further clinical decline with the inability to follow commands, demonstrated cerebral cerebritis and obstructive hydrocephalus requiring transfer to the Neurosurgical ICU and placement of an extraventricular drain (Fig. 1b). CSF analysis demonstrated lymphocytic pleocytosis (89% lymphocytes), along with elevated protein and normal glucose, consistent with a viral infection (Table 1). CSF serology was negative for Powassan virus IgM antibodies (ELISA) but positive for Powassan virus by metagenomic next-generation sequencing (performed at University of California—San Francisco (UCSF) Medical Center Clinical Laboratories). This patient had a longstanding history of hypogammaglobulinemia, requiring chronic IVIG at 6-week intervals to maintain IgG in the 400–600 mg/dL range. On admission, IgG was 416 mg/dL (Table 1). The falsely negative serology was attributed to hypogammaglobulinemia, and he was diagnosed with Powassan virus encephalitis. Throughout the prolonged hospitalization, the patient did not demonstrate meaningful neurologic improvement, and he was transitioned to comfort care.

Since its original description in 1958, 206 cases of neuroinvasive Powassan virus infections have been reported in the U.S. through the end of 2020 [4–6]. Cases in humans have risen 20–30-fold from the 1990s to the late 2010s, increasing from an estimated 1 case per year to 20–30 cases per year. Neuroinvasive disease due to the Powassan virus is a rare but serious complication. Unlike rickettsial illnesses, the transmission of the Powassan virus can occur within 30 min of tick attachment. The incubation time from tick bite to symptomatic disease ranges from 1 to 4 weeks, with initial symptoms including fever, headache, vomiting, and weakness (Supplemental Fig. 1). Symptoms of severe disease include confusion, seizures, depressed level of consciousness, or focal neurologic findings (loss of coordination, difficulty speaking), can appear very similar to herpes encephalitis [4]. The majority of those infected in the general population will experience no symptoms, as evidenced by up to 5% seropositivity of individuals in endemic regions [7]. Currently, there is no effective antiviral treatment for Powassan virus infection. There have been promising vaccine developments that have not yet made their way to human testing [8]. Powassan virus has been isolated from a variety of ticks, including *Ixodes scapularis*, other *Ixodes* species, and *Dermacentor andersoni*. The incidence of neuroinvasive Powassan virus in the United States is consistent with the geographic distribution of these ticks, with concentrations in the Upper Midwest and Northeast [4].

Defects in humoral and cellular immunity due to CLL itself and its treatment result in an increased risk of infections that are a significant cause of morbidity and mortality in CLL patients [9]. Most reports of opportunistic infections in CLL patients are limited to case reports or small institutional case series. The few large cohort studies that investigated opportunistic infections in CLL patients describe a variety of infections. A Swedish study in the pre-ibrutinib era described 829 inpatient opportunistic infections (the most common being Pneumocystis, Herpes zoster, Pseudomonas, Candida, and Aspergillus) in ~9000 CLL patients with a median follow-up of more than five years [10]. An Italian study of 494 patients treated in the ibrutinib era reported pneumonia, grade ≥3 non-opportunistic infections, and opportunistic infections in 32% of patients with an overall incidence rate per 100 person-year of 15.3% [11]. Herpes virus reactivation has been described with CLL treatments such as ibrutinib and idelalisib, and antiviral prophylaxis appears effective [12]. Of note, both patients in this report had been previously treated with chemoimmunotherapy, including pentostatin, cyclophosphamide, and rituximab, with the last exposure to treatment 9 and 13 years ago, respectively. In addition, these patients were on ibrutinib therapy at the time of their current clinical presentation, and one of the two patients required IVIG replacement therapy for significant hypogammaglobulinemia—suggesting that the etiology of the Powassan virus encephalitis was likely multifactorial. These studies highlight the importance of continued vigilance for such infections in patients who have been extensively treated for CLL and are receiving novel agents such as ibrutinib.

To the best of our knowledge, this represents the first report of fatal Powassan virus encephalitis among CLL patients. There is a case report of a single patient with CLL, not on treatment, who contracted Powassan encephalitis and survived, although he experienced significant morbidity, including aphasia and spastic paraplegia [13]. Other reports of neuroinvasive Powassan virus among the immunocompromised include a 67-year-old female receiving adjuvant chemotherapy following hemicolectomy [14], a 61-year-old male receiving adalimumab for Crohn’s disease [15], and a 63-year-old male with follicular lymphoma treated with rituximab [16].

Due to their uniquely immunocompromised state, CLL patients are at increased risk of serious infection, and the community of providers treating CLL should be aware of this pathogen. As seen in these cases, many CLL patients develop hypogammaglobulinemia, which only further worsens infectious risk. Educating patients residing in or visiting areas where the Powassan virus is endemic about strategies to mitigate tick bites is paramount in preventing infection. This includes the application of insect repellents to the skin (for example, 25–35% DEET for adults) plus permethrin insecticide-treated clothing.

The lack of a widely available molecular test may hamper the detection of the Powassan virus early in its course. Current testing options include IgM ELISA serologies (confirmed with plaque reduction assays), reverse-transcriptase PCR (RT-PCR) testing of serum, CSF, or tissue samples, immunohistochemistry testing of tissue, and metagenomic next-generation sequencing. Furthermore, for patients with CLL or other causes of immunocompromise in which Powassan virus encephalitis is in the differential, it is important to understand that they may be unable to mount an antibody response, potentially resulting in false negative Powassan virus IgM serologies. Such was the case with one of our patients and has been reported in other clinic contexts [17]. Metagenomic next-generation sequencing, available from UCSF, and PCR testing, available at the CDC and some state public health labs, have emerged as useful tools for diagnosing Powassan virus, and clinicians may consider deploying if there is a high index of suspicion in the face of negative serologic assays [16, 17].

In summary, we report two patients with high-risk CLL who were on ibrutinib therapy and developed worsening neurological symptoms following a tick bite. The constellation of clinical presentation, MRI findings, and CSF lymphocytic pleocytosis in the absence of malignant cells led to a diagnosis of viral encephalitis. Further CSF studies confirmed a diagnosis of Powassan virus encephalitis—which despite maximal supportive care, led to our patients’ deaths.

These cases illustrate the importance of keeping Powassan virus encephalitis in the differential diagnosis of CLL patients who reside in the Midwest, and other endemic areas of this infection, and who present with signs and symptoms of encephalitis. In the absence of definitive therapy and given poor outcomes following Powassan virus infection, adherence to CDC guidelines to prevent tick bites (https://www.cdc.gov/ticks/avoid/on_people.html) is of utmost importance.

## Supplementary information

Supplemental Figure 1.

Supplemental Figure Legend

## Data Availability

Data presented in this article are available via email request from corresponding author.
