# Supplementary figures and images for: Fatal Powassan virus encephalitis in patients with chronic lymphocytic leukemia
Source: Blood Cancer J. 2022 Oct 7;12(10):143. doi: 10.1038/s41408-022-00737-y (PMC9537528; doi:10.1038/s41408-022-00737-y)

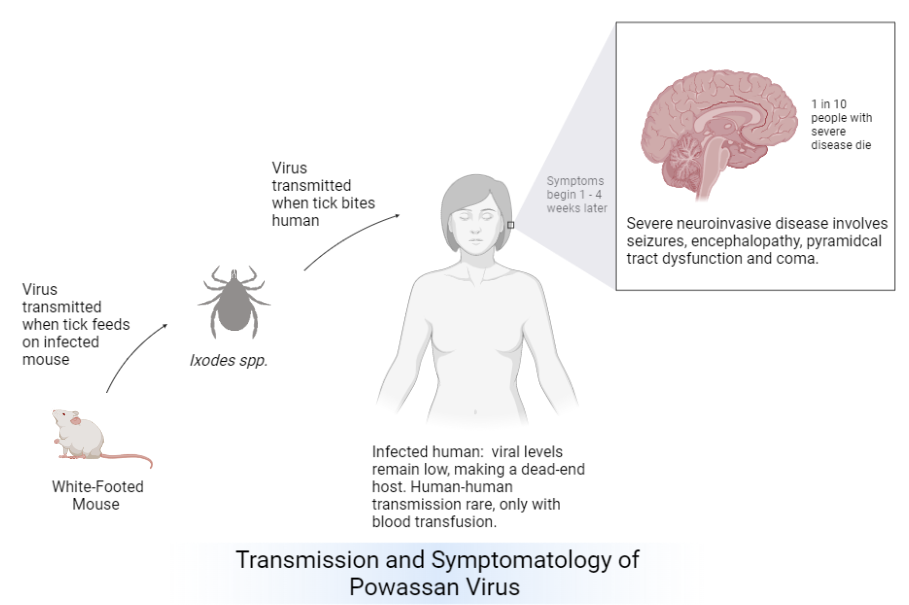

Supplement: Supplementary file 1 — Supplemental Figure 1. [file 41408_2022_737_MOESM1_ESM.docx]
