# Supplementary material for: Fatal Powassan virus encephalitis in patients with chronic lymphocytic leukemia
Source: Blood Cancer J. 2022 Oct 7;12(10):143. doi: 10.1038/s41408-022-00737-y (PMC9537528; doi:10.1038/s41408-022-00737-y)
Supplement: Supplementary file 2 — Supplemental Figure Legend [file 41408_2022_737_MOESM2_ESM.docx]

Supplemental Figure Legend: Supplemental Figure 1. Transmission and symptomatology of the Flavivirus Powassan Virus.
